# Machine learning-assisted screening for canine Cushing’s syndrome

**DOI:** 10.1080/01652176.2025.2604643

**Published:** 2025-12-22

**Authors:** Young-Jae Yoo, Kyungchang Jeong, Hanbit Seo, Ha-Suk Nam, Yeon Chae, Taesik Yun, Byeong-Teck Kang, Euijong Lee, Hakhyun Kim

**Affiliations:** a Laboratory of Veterinary Internal Medicine, College of Veterinary Medicine, Chungbuk National University, Cheongju, Republic of Korea; b School of Computer Science, Chungbuk National University, Cheongju, Republic of Korea

**Keywords:** gradient boosting, boosted tree, artificial intelligence, dog, hypercortisolism, non-adrenal illness, polydipsia, polyuria

## Abstract

Cushing’s syndrome (CS) is a common endocrine disorder in dogs that can significantly impair their quality of life. Diagnosis is often challenging because of its variable clinical presentation, making it difficult to identify suitable candidates for further diagnostic tests. This study employed machine learning algorithms to assist in CS diagnosis using routinely available screening diagnostics, including complete blood count, serum chemistry panel, and urinalysis parameters such as urine specific gravity and urine protein-to-creatinine ratio. Data were collected from 153 control dogs initially suspected of CS but later excluded and 152 dogs with confirmed CS. A boosted tree algorithm (gradient boosting) was trained on 80% of the collected data, with the remaining 20% reserved for testing. The developed model demonstrated an accuracy of 88.5% [95% confidence interval (CI): 80.5–96.5%], a sensitivity of 83.3% (95% CI: 70.7–96.7%), a specificity of 93.5% (95% CI: 84.9–100%), and an area under the receiver operating characteristic curve of 0.912 (95% CI: 0.835–0.988), indicating excellent discriminatory ability. A user-friendly graphical interface was also developed to facilitate clinical implementation, potentially improving diagnostic efficiency and owner satisfaction.

## Introduction

Cushing’s syndrome (CS) is a common endocrine disorder in dogs caused by chronic excess circulating glucocorticoids (Behrend et al. 2013). This hormonal imbalance leads to classic clinical signs, such as polyuria, polydipsia, polyphagia, a potbellied appearance, muscle weakness, bilateral alopecia, panting, and lethargy. However, some dogs exhibit subtle or limited signs, making the diagnosis more challenging (Behrend et al. 2013; Nagata et al. 2017; Schofield et al. 2020). These clinical signs, along with complications, such as diabetes mellitus, pancreatitis, and hypertension, necessitate timely diagnosis and effective management to enhance the quality of life of affected animals (Miceli et al. 2017; Carotenuto et al. 2019). Despite its importance, diagnosing CS remains challenging owing to its non-specific clinical features, low prevalence (0.28% in the general canine population), and the absence of a highly accurate diagnostic test (Behrend et al. 2013; Bennaim et al. 2018; Schofield et al. 2019; Carvalho et al. 2025). Ensuring an accurate and timely diagnosis of CS is crucial for initiating appropriate treatment early (Peterson 2007). Conversely, misdiagnosis may result in unnecessary treatment, posing potential risks to the patient. Therefore, the development of more comprehensive and reliable diagnostic methods for CS is essential for veterinary practice.

The adrenocorticotropic hormone (ACTH) stimulation test, low-dose dexamethasone suppression test (LDDST), and urine cortisol-to-creatinine ratio (UCCR) test are commonly used screening tests for CS in veterinary medicine (Peterson 2007). However, one study reported that in some European countries, up to 60% of primary care veterinarians conduct screening tests even in dogs without overt clinical signs of CS, raising concerns about diagnostic errors and overdiagnosis (Carvalho et al. 2025). Such indiscriminate use of screening tests may lead to unnecessary confirmatory testing, increased financial burdens on pet owners, and inappropriate treatment of false-positive cases. Accurate screening is essential for distinguishing actual cases from those with overlapping clinical signs or laboratory abnormalities caused by other underlying conditions. Advanced statistical approaches and machine learning models have emerged as promising alternatives to conventional prediction methods for improving diagnostic accuracy and reducing unnecessary testing (Weng et al. 2017). These approaches may be helpful in screening for CS in dogs.

Machine learning, a branch of artificial intelligence, has surpassed traditional risk models in disease prediction by effectively capturing complex non-linear relationships among variables and managing numerous features (Weng et al. 2017; Christodoulou et al. 2019). These algorithms have been increasingly documented in both human and veterinary medical research and have been applied to various clinical challenges, including the use of laboratory data to diagnose Addison’s disease in dogs and chronic kidney disease in cats (Cihan et al. 2017; Weng et al. 2017; Bradley et al. 2019; Reagan et al. 2020). One study demonstrated the effectiveness of machine learning-based models in predicting CS using clinical data, including signalment (age, breed, and sex) and clinical signs (polyuria, polydipsia, alopecia, muscle weakness, and lethargy), indicating their promising diagnostic potential (Schofield et al. 2021). A machine learning model for predicting CS in dogs could assist veterinarians in clinical practice by supporting timely diagnosis and early initiation of appropriate treatment in affected dogs. However, no prior studies have applied machine learning models using laboratory test parameters for CS diagnosis.

This study is the first to develop and internally validate a gradient boosting-based machine learning algorithm that estimates the likelihood of CS from routinely obtained laboratory data, including hematologic, biochemical, and urinalysis parameters, thereby providing an objective and standardized method for assessing the need for confirmatory testing. We hypothesized that a model relying solely on objective laboratory measures would deliver clinically meaningful discrimination and calibration, offering a practical pre-test triage tool to improve prioritization for confirmatory endocrine testing in dogs with suspected CS.

## Materials and methods

### Dataset

A search was conducted using electronic medical records of dogs evaluated for CS at Chungbuk National University Veterinary Teaching Hospital between 2010 and 2024. Inclusion criteria were complete laboratory profiles, including complete blood count (CBC), serum chemistry, urine specific gravity (USG), and urine protein-to-creatinine ratio (UPC). Additionally, dogs were included if they exhibited clinical suspicion of CS, defined by at least two characteristic clinical signs, including panting, polyuria, polydipsia, polyphagia, bilateral alopecia, abdominal distention, poor hair coat, or thin skin (Kim et al. 2019).

Dogs were excluded if they had received CS treatments, including trilostane or mitotane, or systemic corticosteroids prior to presentation or if two or more of the following biochemical parameters were unavailable: alkaline phosphatase (ALP), cholesterol (CHOL), or triglyceride (TG).

All included dogs underwent physical examination, CBC (ProCyte Dx Analyzer^®^, IDEXX Laboratories, Inc., Westbrook, ME, USA), serum biochemistry (Hitachi 7020, Hitachi High-Technologies Co., Tokyo, Japan), and urinalysis, which included USG (PAL-USG, ATAGO Co., Ltd., Tokyo, Japan) and UPC (Catalyst One, IDEXX Laboratories, Westbrook, ME, USA). Routine abdominal radiography and ultrasonography were also performed.

### CS group

The dogs were classified into the CS group based on hormonal and imaging criteria. CS was diagnosed if at least one hormonal test was positive, including either an ACTH stimulation test or an LDDST, combined with ultrasonographic evidence of adrenal gland enlargement. For the ACTH stimulation test, synthetic ACTH (Synacthen; Novartis, Basel, Switzerland; 250 μg/dog) was administered intravenously, and serum cortisol concentrations were measured before and 1 h after the injection. A post-ACTH cortisol concentration >22 µg/dL was interpreted as positive. For the LDDST, dexamethasone (Jeil Pharm Co., Daegu, South Korea; 0.01 mg/kg IV) was administered, and serum cortisol concentrations were measured at baseline, 4, and 8 h using a chemiluminescent immunoassay. The cortisol assay was performed using Immulite 1000 (Siemens Healthcare Diagnostics Ltd., Gwynedd, UK) before 2015 and Immulite 2000 thereafter. An 8-h cortisol concentration >1.5 µg/dL was considered diagnostic for CS (Rodríguez Piñeiro et al. 2011; Bennaim et al. 2018).

Ultrasonographic findings for CS included adrenal gland enlargement, defined as bilateral enlargement (right adrenal diameter >0.60 cm and left adrenal diameter >0.62 cm in dogs weighing <12 kg, or >0.72 and >0.70 cm, respectively, in dogs weighing >12 kg) or unilateral enlargement/mass consistent with adrenal-dependent hypercortisolism (Bento et al. 2016). These findings were interpreted as supportive evidence and were not used as a sole exclusion criterion; imaging results were evaluated in conjunction with endocrine testing and clinical context.

### Control group

Dogs in the control group were initially suspected of having CS but were later determined to have non-adrenal illness based on endocrine test results and clinical follow-up. These dogs either had an identifiable alternative diagnosis explaining the presenting clinical signs, exhibited spontaneous resolution of symptoms without treatment, or displayed normal adrenal gland dimensions on ultrasonography. Imaging findings were interpreted alongside endocrine testing and clinical follow-up.

All control dogs tested negative for CS on hormonal testing, including an LDDST with 8-h cortisol concentrations <1.5 µg/dL and/or a UCCR <31 (Rodríguez Piñeiro et al. 2011; Behrend et al. 2013). Accordingly, the control population comprised dogs in whom CS was excluded and whose subsequent clinical course was not consistent with CS. These cases either had a documented non-adrenal diagnosis explaining the presenting signs or, in the absence of a specific alternative diagnosis, showed resolution or non-progression of signs on follow-up. The 152 control dogs were summarized into the following diagnostic categories: renal–urinary, 41/152 (27.0%; e.g. chronic kidney disease, protein-losing nephropathy, bacterial cystitis); endocrine–metabolic (non-adrenal) 37/152 (24.3%; e.g. diabetes mellitus, hypothyroidism, hyperlipidemia/dyslipidemia); neoplasia (including pheochromocytoma and non-functional adrenal tumor) 25/152 (16.4%); hepatobiliary 20/152 (13.2%; e.g. vacuolar hepatopathy, chronic hepatitis, cholangiohepatitis, cholestatic diseases including gallbladder mucocele); undiagnosed 25/152 (16.4%; CS excluded hormonally with subsequent resolution or non-progression of signs); gastrointestinal–pancreatic 3/152 (2.0%; e.g. pancreatitis and chronic gastritis); and cardiopulmonary 1/152 (0.7%; right-sided congestive heart failure).

### Machine learning

**Figure 1.**
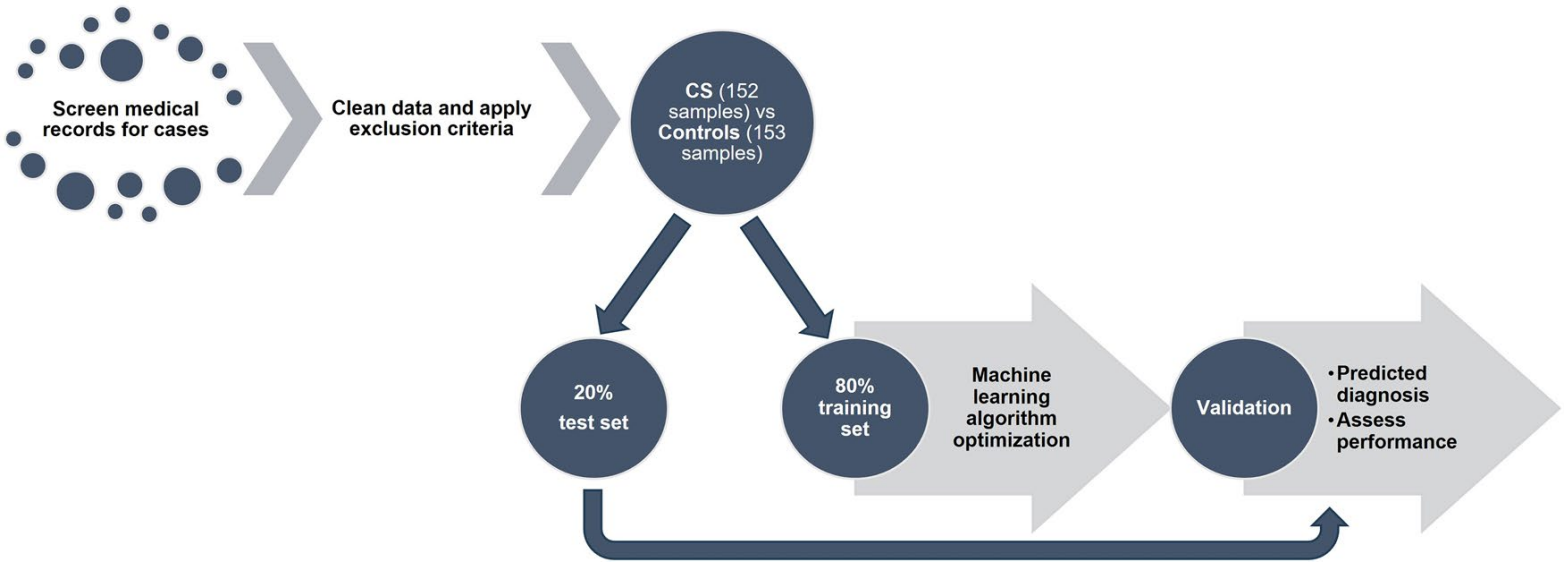
Experimental design.

The input features of the machine learning model included routinely collected hematological, biochemical, and urinalysis parameters (Table 1). Dogs with partially incomplete laboratory datasets were included in the analysis, and missing values were addressed during the data preprocessing phase through imputation and feature scaling (Ahsan et al. 2021). The dataset was randomly divided, with 80% allocated to the training set and the remaining 20% to the test set to evaluate model performance (Figure 1).

**Table 1.** Input parameters.

CBC	Serum chemistry	Urinalysis
WBC (×10^3^/μL)	Total protein (g/dL)	USG
Monocyte (×10^3^/μL)	Albumin (g/dL)	UPC
Lymphocyte (×10^3^/μL)	Globulins (g/dL)	
Neutrophil (×10^3^/μL)	ALT (IU/L)	
Eosinophil (×10^3^/μL)	ALP (IU/L)	
Basophil (×10^3^/μL)	Total bilirubin (mg/dL)	
PCV (%)	BUN (mg/dL)	
Hemoglobin (g/dL)	Creatinine (mg/dL)	
MCV (fL)	CHOL (mg/dL)	
MCHC (g/dL)	TG (mg/dL)	
Platelets (×10^3^/μL)	Glucose (mg/dL)	
PLR	Sodium (mmol/L)	
NLR	Potassium (mmol/L)	
	Chloride (mmol/L)	
	Calcium (mg/dL)	
	Phosphorus (mmol/L)	

Blood work features used as input for machine learning algorithms.

ALP, alkaline phosphatase; ALT, alanine transaminase; BUN, blood urea nitrogen; CBC, complete blood count; CHOL, cholesterol; MCHC, mean corpuscular hemoglobin concentration; MCV, mean corpuscular volume; NLR, neutrophil-to-lymphocyte ratio; PCV, packed cell volume; PLR, platelet-to-lymphocyte ratio; TG, triglyceride; UPC, urine protein-to-creatinine ratio; USG, urine specific gravity; WBC, white blood cell.

Gradient boosting was selected as the classification algorithm owing to its capacity to handle complex non-linear relationships in clinical data (Friedman 2001; Kennedy et al. 2023). To prevent overfitting, hyperparameter tuning was performed using Bayesian optimization combined with five-fold cross-validation. A maximum of 500 decision trees was allowed during model construction. The final model, selected based on the highest classification accuracy in the training data, comprised 93 weak learners.

### Statistical analyses

All statistical analyses were performed using commercially available software (Prism 9; GraphPad Software Inc., San Diego, CA, USA). The normality of continuous variables was assessed using the Shapiro–Wilk test. As none of the continuous variables followed a normal distribution, quantitative data are expressed as medians with interquartile ranges (IQRs), and the Mann–Whitney *U* test was applied for univariate comparisons between the CS and control groups. Categorical variables were analyzed using Pearson’s chi-squared test or Fisher’s exact test, as appropriate. The significance level of *P* < 0.05 was considered statistically significant for all analyses.

Model performance was evaluated using standard classification metrics, including accuracy, sensitivity, specificity, and area under the curve (AUC), with all values reported along with 95% confidence intervals (CIs). These metrics were derived from the confusion matrix of the test set predictions. Receiver operating characteristic (ROC) curve analysis was conducted using Prism 9, and the optimal decision threshold was determined using Youden’s index. All machine learning procedures, statistical analyses, and graphical user interface development were performed using MATLAB software (MathWorks, Natick, MA, USA).

## Results

### Demographic information

A total of 305 dogs met the inclusion criteria and were included in the analysis: 153 dogs in the CS group and 152 in the control group. The median age was 12 years (IQR: 10–14) in the CS group and 11 years (IQR: 9–13) in the control group (*P* = 0.045). However, there were no significant differences in sex distribution (*P* = 0.731) or body weight (*P* = 0.069) between the two groups. Twenty-three different breeds were represented in the CS group and 27 in the control group. The three most common breeds were Maltese (46/153 in the CS group vs. 36/152 in the control group, *P* = 0.20), Shih Tzu (36/153 vs. 25/152, *P* = 0.12), and Yorkshire Terrier (15/153 vs. 14/152, *P* = 0.85), with no statistically significant differences in breed distribution between the groups. Detailed demographic characteristics are presented in Table 2.

**Table 2.** Population demographics.

Characteristics	Control group	CS group	*P* value
Sample size	152	153	–
Male sex (*n*, %)	71 (47%)	75 (49%)	0.731
Weight (kg)	5.26 (4.06–7.50)	5.10 (3.73–6.58)	0.069
Age (years)	11 (9–13)	12 (10–14)	0.045*

Data given as median (IQR). Mann–Whitney *U* test was applied. Data given as numbers. Pearson’s chi-squared test or Fisher’s exact test was applied.

CS, Cushing’s syndrome.

*P value < 0.05, statistically significant.

### Clinicopathological results

All dogs included in the study had CBC, serum chemistry, and urinalysis data. Table 3 summarizes the clinicopathological results. Compared to controls, dogs with CS showed higher leukocyte counts, increased platelet-to-lymphocyte ratio and neutrophil-to-lymphocyte ratio values, elevated liver enzyme and lipid concentrations, and abnormal urinalysis findings. Notably, ALP, CHOL, TG, and glucose concentrations were significantly higher in the CS group, whereas USG was lower and UPC was higher than those in controls.

**Table 3.** Clinicopathologic values.

Characteristics	Control group^a^	CS group^a^	*P* value
WBC (×10^3^/μL)	8.8 (7.0–11.9)	10.7 (8.7–14.4)	<0.0001*
Monocyte (×10^3^/μL)	0.47 (0.33–0.65)	0.71 (0.44–1.14)	<0.0001*
Lymphocyte (×10^3^/μL)	1.7 (1.3–2.3)	1.4 (1.0–2.0)	0.0004*
Neutrophil (×10^3^/μL)	5.8 (4.6–8.3)	8.1 (6.1–12.0)	<0.0001*
Eosinophil (×10^3^/μL)	0.3 (0.21–0.49)	0.2 (0.09–0.35)	<0.0001*
Basophil (×10^3^/μL)	0.02 (0.01–0.04)	0.016 (0–0.04)	0.2070
PCV (%)	46.0 (42.2–50.2)	44.0 (38.2–48.8)	0.0064*
Hemoglobin (g/dL)	16.2 (14.9–17.6)	15.5 (13.4–17.2)	0.0038*
MCV (fL)	64.3 (61.9–66.7)	66.0 (62.5–68.6)	0.2016
MCHC (g/dL)	34.9 (34.5–35.9)	35.0 (34.2–35.9)	0.4364
Platelets (×10^3^/μL)	414 (332–515)	504 (374–629)	<0.0001*
PLR	225.2 (170.2–331.7)	356.3 (228.6–559.0)	<0.0001*
NLR	3.35 (2.62–4.69)	6.05 (3.64–9.36)	<0.0001*
Total protein (g/dL)	6.6 (6.1–7.0)	6.4 (5.9–6.9)	0.0561
Albumin (g/dL)	3.0 (2.7–3.3)	2.95 (2.7–3.3)	0.3614
Globulins (g/dL)	3.5 (3.2–3.9)	3.4 (3.1–3.8)	0.1311
ALT (IU/L)	58 (34–92)	117 (59–378)	<0.0001*
ALP (IU/L)	491 (205–1109)	1549 (691–3063)	<0.0001*
Total bilirubin (mg/dL)	0 (0–0.02)	0 (0–0.1)	0.2990
BUN (mg/dL)	18.1 (13.4–28.4)	18.7 (12.6–31.9)	0.6481
Creatinine (mg/dL)	0.82 (0.70–1.1)	0.80 (0.60–1.04)	0.9270
CHOL (mg/dL)	240 (199–292)	318 (232–417)	<0.0001*
TG (mg/dL)	89 (62–146)	146 (97–228)	0.0013*
Glucose (mg/dL)	111 (102–123)	115 (102–134)	0.0178*
Sodium (mmol/L)	147 (145–148)	146 (144–149)	0.7546
Potassium (mmol/L)	5.0 (4.6–5.3)	5.0 (4.6–5.4)	0.3805
Chloride (mmol/L)	113 (111–116)	111 (108–114)	0.0030*
Calcium (mg/dL)	9.8 (9.2–10.5)	9.8 (9.1–10.40)	0.5603
Phosphorus (mmol/L)	3.6 (2.9–4.2)	4.0 (3.3–4.8)	0.0001*
USG	1.023 (1.014–1.034)	1.019 (1.013–1.026)	0.0068*
UPC	0.27 (0.12–0.79)	0.61 (0.25–1.74)	0.0003*

Mann–Whitney *U* test was applied.

ALP, alkaline phosphatase; ALT, Alanine transaminase; BUN, blood urea nitrogen; CHOL, cholesterol; CS, Cushing’s syndrome; MCHC, mean corpuscular hemoglobin concentration; MCV, mean corpuscular volume; NLR, neutrophil-to-lymphocyte ratio; PCV, packed cell volume; PLR, platelet-to-lymphocyte ratio; TG, triglyceride; UPC, urine protein-to-creatinine ratio; USG, urine specific gravity; WBC, white blood cell.

^a^ Values reported are median (IQR).

*P value < 0.05, statistically significant.

### Classification test and algorithm performance

Of the 305 dogs, 244 (80%) were randomly allocated to the training set, and 61 (20%) to the test set. A gradient boosting algorithm was used to construct a classification model using routinely collected clinicopathological data. The performance of the trained model was evaluated during both the training and testing phases. As summarized in Table 4, the model demonstrated excellent performance on the training set, achieving an accuracy of 0.959 (95% CI: 0.934–0.984), sensitivity of 0.943 (CI: 0.901–0.984), specificity of 0.975 (CI: 0.948–1.000), and AUC of 0.993 (CI: 0.983–1.000). In the testing dataset, the model retained strong generalizability, attaining an accuracy of 0.885 (CI: 0.805–0.965), sensitivity of 0.833 (CI: 0.700–0.967), specificity of 0.935 (CI: 0.849–1.000), and AUC of 0.912 (CI: 0.835–0.988). The reduced model (excluding both USG and UPC) maintained performance comparable to the full model: accuracy of 88.5% (54/61), sensitivity of 83.3% (25/30), specificity of 93.5% (29/31), positive predictive value of 92.6% (25/27), negative predictive value of 85.3% (29/34), and AUC of 0.905 (Supplementary Figure S1). When only UPC was excluded, performance remained clinically useful, with accuracy of 86.9% (53/61), sensitivity of 83.3% (25/30), specificity of 90.3% (28/31), positive predictive value of 89.3% (25/28), negative predictive value 84.8% (28/33), and AUC of 0.917 (Supplementary Figure S2).

**Table 4.** Performance metrics of the gradient boosting model on training and testing datasets.

Metric	Gradient boosting
*Training dataset performance measures*	
Accuracy	0.959 (0.934–0.984)
Sensitivity	0.943 (0.901–0.984)
Specificity	0.975 (0.948–1.000)
AUC	0.993 (0.983–1.000)
*Testing dataset performance measures*	
Accuracy	0.885 (0.805–0.965)
Sensitivity	0.833 (0.700–0.967)
Specificity	0.935 (0.849–1.000)
AUC	0.912 (0.835–0.988)

Values are presented as point estimates with 95% CIs.

AUC, area under the ROC curve.

**Figure 2.**
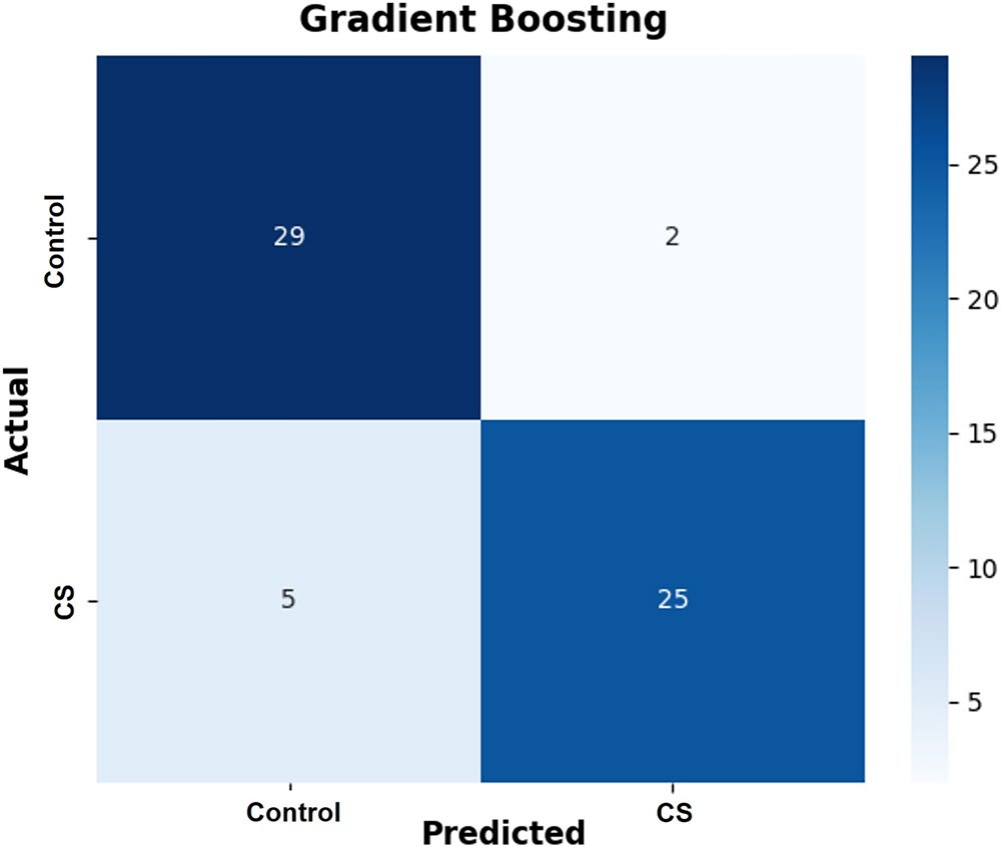
Confusion matrix illustrating the classification performance of the gradient boosting model.

The confusion matrix in Figure 2 illustrates the classification outcomes, displaying correct and incorrect predictions for each group. Notably, only two control dogs (2/152, 1.3%) were misclassified as having CS, underscoring the model’s high specificity, which is clinically important for minimizing false-positive results. Follow-up information for these dogs was not available, and their final diagnoses could not be confirmed.

**Figure 3.**
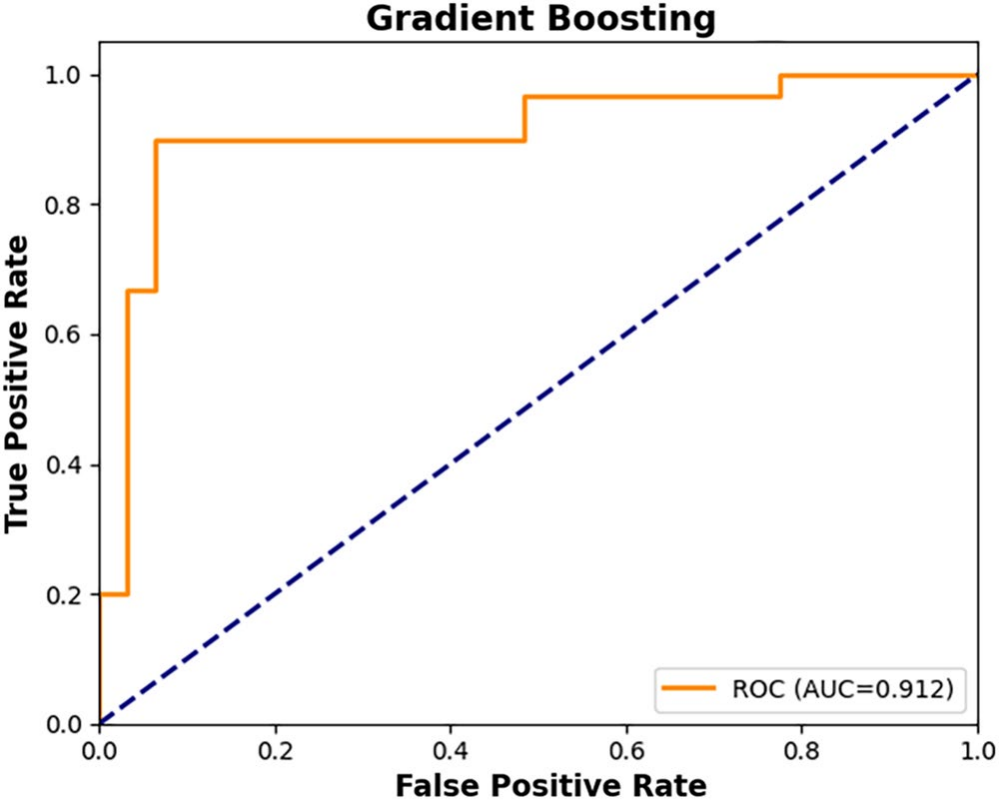
ROC curve showing model performance on the test dataset.

The ROC curve for the test data is shown in Figure 3, demonstrating strong discriminative capacity across multiple thresholds.

### Construction of the user interface

**Figure 4.**
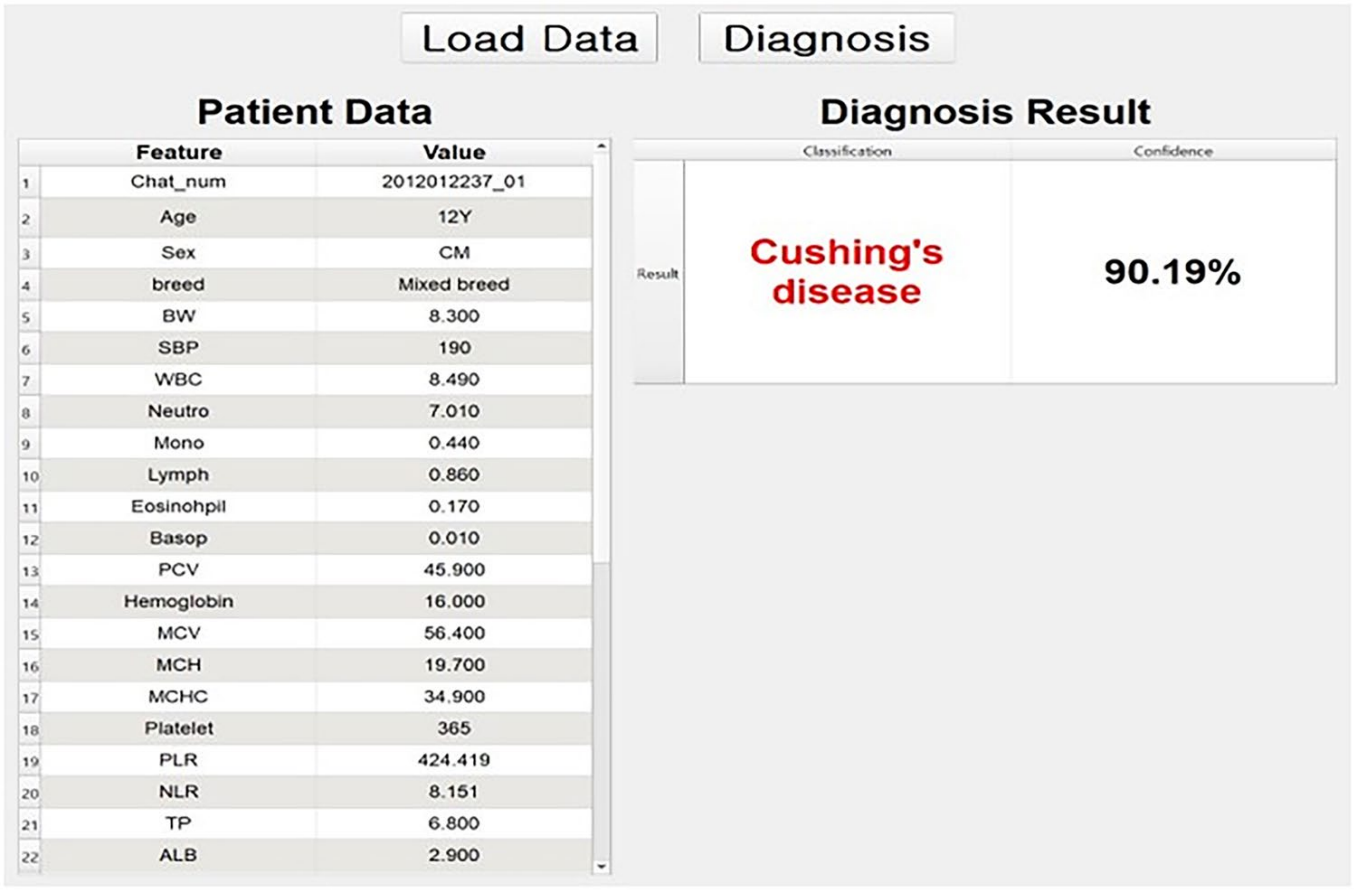
Representative application of the machine learning model in predicting hypercortisolism in an individual dog.

A graphical user interface was developed to enhance clinical applicability and implement the trained machine learning model for point-of-care usage. As shown in Figure 4, the interface allows users to input patient variables and provides a binary classification result and confidence score. In a representative case, a 12-year-old mixed-breed castrated male dog was classified as ‘CS Suspected’ with a prediction confidence of 90.19%. This tool is designed to assist veterinarians in identifying candidates for further confirmatory hormone testing.

## Discussion

In this study, a gradient boosting-based machine learning algorithm was developed and validated for predicting CS in dogs using routinely available laboratory data. The model demonstrated high diagnostic performance, achieving 88.5% accuracy, 83.3% sensitivity, 93.5% specificity, and an AUC of 0.912 in the test dataset. Applying this machine learning approach to standard clinicopathological parameters, such as CBC, serum chemistry, and urinalysis, allows the likelihood of CS to be estimated prior to confirmatory hormonal testing. Furthermore, performance remained clinically useful even whether UPC and/or USG were excluded. Consequently, the model is intended to be a clinical decision-support tool to assist veterinarians in determining which patients require further diagnostic evaluation, potentially enhancing diagnostic efficiency and reducing unnecessary confirmatory testing.

Accurate diagnosis of CS is clinically significant because of its substantial influence on patient prognosis and the potential adverse effects associated with inappropriate treatment. Screening for CS in primary care veterinary practice is often initiated based on non-specific clinical presentations, increasing the risk of false-positive diagnoses and inappropriate therapeutic interventions (Carvalho et al. 2025). For example, administering trilostane without a definitive diagnosis can result in iatrogenic hypoadrenocorticism, with severe clinical consequences (King and Morton 2017). Additionally, timely and accurate diagnosis is particularly important in pituitary-dependent hypercortisolism, where survival outcomes can vary depending on treatment initiation (Nagata et al. 2017). Therefore, the systematic and accurate identification of dogs that require further endocrine evaluation is clinically important.

Conventional endocrine tests typically used to diagnose CS include the ACTH stimulation test, LDDST, and UCCR. However, their diagnostic performance varies: LDDST offers 85–100% sensitivity but only 44–73% specificity; the ACTH stimulation test shows 57–61% sensitivity and 59–93% specificity; and UCCR has high sensitivity (97%) but low specificity (∼51%) (Monroe et al. 2012; Nivy et al. 2018), implying that no tests are the gold standard to diagnose CS in dogs. Although these endocrine tests remain essential components of diagnostic protocols, limitations exist, such as high costs, prolonged test duration, patient stress due to serial blood sampling or hospitalization, and decreased specificity in dogs with concurrent non-adrenal illnesses. Conversely, the machine learning model presented in the present study achieves a balanced diagnostic performance while relying on objective laboratory parameters routinely available in veterinary clinics, thereby addressing some of these limitations. Because the clinical diagnosis of CS depends on the LDDST and the ACTH stimulation test, we defined case/control labels according to these reference standards. Accordingly, the proposed algorithm is intended as a pre-test triage aid rather than a replacement for endocrine testing.

Endocrine test cut-offs and analytical platforms vary among institutions. In this study, relatively conservative thresholds were applied using local reference intervals to prioritize label specificity during model development. As a result, the model is expected to perform most accurately under comparable testing conditions, and local recalibration may be warranted when different platforms or thresholds are used. Ultrasonography is also operator dependent; normal adrenal dimensions do not exclude CS, and adrenal enlargement is not pathognomonic.

Borderline endocrine results (e.g. UCCR 20–30; LDDST near 1.0–1.5 μg/dL) should be considered indeterminate and integrated in the context of clinical assessment and follow-up testing, rather than used for definitive classification. Importantly, a negative model prediction should not preclude confirmatory hormonal testing when clinical suspicion is high, as the algorithm is designed to support pre-test prioritization rather than replace confirmatory evaluation. A tiered reference framework (positive/indeterminate/negative) may be valuable in future prospective validation.

The diagnostic performance of the current model compares favorably with previously reported studies. A logistic regression model reported by Schofield et al. (2021), which utilized subjective clinical data, including owner-reported signs and physical examination findings, achieved an AUC of 0.85 and an accuracy of 77%. The model developed in this study demonstrated superior diagnostic performance using exclusively objective numerical laboratory data. This outcome emphasizes the reliability and advantages of structured numerical data in veterinary diagnostic modeling, particularly in retrospective analyses, owing to the decreased susceptibility to bias and variability inherent in subjective assessments. However, the cost of performing laboratory tests could limit the utility of the current model compared to the previous logistic regression model reported by Schofield et al.

The laboratory abnormalities observed in this study are consistent with the findings described in the ACVIM consensus guidelines for CS in dogs. Dogs diagnosed with CS frequently demonstrate increased ALP, CHOL, glucose, and neutrophil concentrations, along with decreased eosinophil and lymphocyte counts (Behrend et al. 2013). Although individual parameters exhibit limited specificity when evaluated separately, integrated analysis through machine learning algorithms enhances diagnostic accuracy and consistency. This study also supports the utility of recently proposed biomarkers, such as the platelet-to-lymphocyte and neutrophil-to-lymphocyte ratios, as described in a previous study, further validating their potential role as supplementary diagnostic indicators in dogs with CS (Yun et al. 2024).

This study has some limitations. Firstly, the model was developed using retrospective data from a single veterinary institution, which may limit its generalizability to other clinical settings. External validity could be affected by variability in breed distribution, regional laboratory reference intervals, and clinical practices among different institutions. Although the sample size of this study is comparable to that of similar veterinary diagnostic modeling studies, it may be insufficient to capture rare or atypical presentations of CS. Future multicenter studies with more diverse populations are needed to strengthen the model’s robustness and enhance its applicability.

Secondly, the model relied on institutional endocrine cut-offs and analytical platforms that were deliberately selected to enhance label specificity; under alternative thresholds, this approach could increase the risk of false-negative labeling. Because relatively conservative thresholds were applied for the ACTH stimulation test, LDDST, and UCCR in accordance with local reference intervals, non-CS cases were more stringently excluded at the labeling stage. Consequently, the model is expected to perform best when used under similar conditions, and local recalibration may be warranted where different platforms or thresholds are applied. Thirdly, ultrasonography was not standardized across operators and examinations, and cross-sectional imaging of the sellar region (computed tomography/magnetic resonance imaging) was not routinely available in this retrospective dataset. Finally, model outputs are intended to complement, not replace, clinical judgment. When clinical suspicion remains high, confirmatory hormonal testing remains indicated, and in selected cases, therapeutic response may be cautiously considered alongside endocrine findings, even when the model prediction is negative.

In conclusion, the gradient boosting-based machine learning algorithm developed in this study demonstrates strong potential as a clinical decision-support tool for identifying dogs who require timely and accurate confirmatory CS testing. Further studies are warranted to evaluate its performance in dogs with early or non-specific clinical signs. By utilizing routinely collected laboratory parameters, the model demonstrated high diagnostic accuracy and was integrated into a user-friendly interface to support clinical utility. Instead of replacing traditional hormonal testing, the model is designed to assist veterinary clinicians in efficiently identifying dogs requiring further endocrine evaluation. Clinical implementation of this algorithm could optimize diagnostic efficiency, facilitate informed therapeutic decisions, and ultimately improve outcomes for dogs with suspected CS.

## Supplementary Material

Supplemental Material

Supplementary Figure S2.tiff

Supplementary Figure S1.tiff

## Data Availability

The datasets generated or analyzed during this study are available from the corresponding author on reasonable request.
