# Supplementary material for: Machine learning-assisted screening for canine Cushing’s syndrome
Source: Vet Q. 2025 Dec 22;46(1):2604643. doi: 10.1080/01652176.2025.2604643 (PMC12777882; doi:10.1080/01652176.2025.2604643)
Supplement: Supplemental Material [file TVEQ_A_2604643_SM7456.docx]

**Supplementary Figure caption**

**Supplementary Figure S1**. Receiver operating characteristic (ROC) curve showing the reduced model (excluding both urine specific gravity and urine protein-to-creatinine ratio) performance on the test dataset. AUC, area under the receiver operating characteristic curve

**Supplementary Figure S2**. Receiver operating characteristic (ROC) curve showing the reduced model (excluding urine protein-to-creatinine ratio) performance on the test dataset. AUC, area v
