# Supplementary figures and images for: Machine learning-assisted screening for canine Cushing’s syndrome
Source: Vet Q. 2025 Dec 22;46(1):2604643. doi: 10.1080/01652176.2025.2604643 (PMC12777882; doi:10.1080/01652176.2025.2604643)

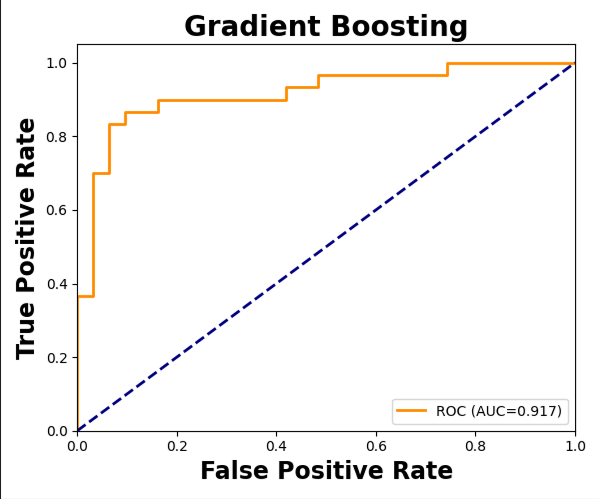

Supplement: Supplementary Figure S2.tiff [file TVEQ_A_2604643_SM3168.tiff]

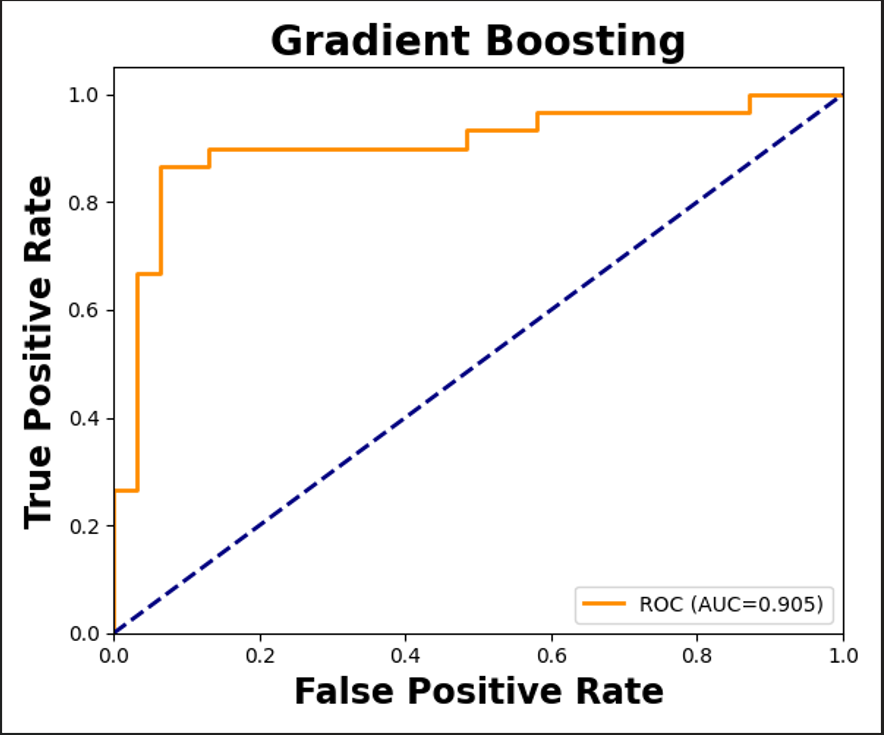

Supplement: Supplementary Figure S1.tiff [file TVEQ_A_2604643_SM3167.tiff]
